# COVID-19: over welke mensen maken we ons extra zorgen?

**DOI:** 10.1007/s12508-020-00290-8

**Published:** 2020-12-18

**Authors:** Marleen M. Kraaij-Dirkzwager, M. U. Yke Tromp, Pieter van der Torn

**Affiliations:** 1 Lectoraat Crisisbeheersing, Instituut Fysieke Veiligheid, Arnhem, Nederland; 2 Vakgroep Geneeskunde, Universiteit Utrecht, Utrecht, Nederland; 3 Itineris, http://www.itineris.nl/

**Keywords:** COVID-maatregelen, publieke gezondheidszorg, kwetsbare groepen, kwetsbaarheden, WHOQOL, crisismanagement, COVID control measures, Public health, Vulnerable groups, Vulnerability, WHOQOL, Crisis management

## Abstract

COVID-19 raakt de samenleving in zijn geheel. Het blijkt lastig om een goede balans te vinden tussen de risico’s van een infectie met SARS-CoV‑2 en de schade door de uitbraakbeheersing (verder: COVID-maatregelen). Meer bescherming voor de één betekent soms meer risico voor het leven, levensonderhoud en samenleven van een ander. Zicht op kwetsbare groepen is nodig om de zorg en ondersteuning te kunnen afstemmen op hun risico’s en behoeften. Dit artikel gaat na welke groepen extra kwetsbaar zijn voor negatieve neveneffecten van COVID-maatregelen. We willen daarmee een discussie op gang brengen over optimale ondersteuning van deze en vergelijkbare groepen mensen tijdens en na deze crisis.

## Abstract (en)

The COVID-19 outbreak and its control measures affect the life, livelihood and way of life of many. Vulnerable groups may require different information, practical, emotional and financial support, depending on their specific vulnerabilities. Early reports for the Dutch government on the impacts of COVID-19 control measures were used for drafting a preliminary overview of vulnerable groups and their vulnerabilities. The overview may serve as first step towards a scoping review and be of use in the professional discussion for targeting and finetuning the support to the groups most in need.

## Aanpak

COVID-maatregelen worden geïmplementeerd om mensen die risico lopen op ernstige COVID te beschermen [1]. In maart tot juni 2020 verschenen diverse rapporten over de neveneffecten van COVID-maatregelen. Artsen Maatschappij en Gezondheid, verenigd in de Denkgroep Publieke Gezondheid rondom COVID-19, selecteerden elf adviesrapporten [2–12]. Criteria voor inclusie waren: a) analyse van COVID-maatregelen vanuit verschillende invalshoeken en b) advies aan de Rijksoverheid. Een geschikte methode voor categorisering van neveneffecten van COVID-maatregelen ontbreekt. Na vergelijking van de Nationale Risicobeoordeling (te algemeen), het positieve gezondheidsconcept en de WHOQOL, kozen we voor exploratie via de WHOQOL [13–15]. De internationaal ontwikkelde WHOQOL brengt de kwaliteit van leven in kaart, is gedetailleerd uitgewerkt en past bij maatschappelijke vraagstukken als de COVID-maatregelen. De zes WHOQOL-domeinen (fysiek, psychisch, zelfstandigheid, sociale relaties, omgeving, spiritualiteit) met 24 (sub)items zijn gebruikt als checklist om (verwachte) neveneffecten van de COVID-maatregelen te inventariseren. Kwetsbare groepen zijn geïnventariseerd aan de hand van de elf adviesrapporten. Een nadere detaillering is beschikbaar in de scriptie die ten grondslag ligt aan dit artikel (op te vragen bij de auteurs). Vergelijking met de internationale COVID-literatuur is vooralsnog niet haalbaar omdat geschikte overzichtsartikelen over kwetsbare groepen ontbreken, afgezien van een vroeg artikel van Douglas et al. [16].

## Resultaten

COVID-19 raakt allen, maar sommigen zijn om verschillende redenen extra kwetsbaar, zoals blijkt uit tab. 1. Zo is een oudere alleenstaande man of vrouw met een chronische aandoening om andere redenen verhoogd kwetsbaar dan een jonge moeder die werkzaam is in de cultuursector en mantelzorger is voor haar ouders. Omwille van de leesbaarheid zijn de WHOQOL-dimensies hieronder herdoopt tot fysieke gezondheid (inclusief toegang tot de zorg), mentale gezondheid, dagelijks leven (in dit verband het belangrijkste subdomein van zelfstandigheid) en sociaal-maatschappelijke omgeving (samenvoeging van sociale relaties en omgeving uit de WHOQOL).

**Table 1:** 

Risicogroepen	Negatieve neveneffecten van COVID-maatregelen
Alleenstaanden (onder wie jongeren, ouderen en chronisch zieken)	Minder sociale contacten → versombering, vereenzaming, minder regie over het eigen leven
Asielzoekers/statushouders	Verlies van regie, minder toekomstperspectief en inactiviteit → versombering; minder participatie in de samenleving
Bewoners van instellingen voor langdurige zorg	Bezoekbeperkingen en minder (geen) dagbesteding → minder informele zorg, versombering, vereenzaming
Dak- en thuislozen	Meer onzekerheid en minder ondersteuning bij per definitie verhoogde kwetsbaarheden door onder andere leefomstandigheden
Ex-gedetineerden	Beperkingen van de participatiemogelijkheden → minder re-integratie/meer terugval
Flexwerkers, zzp’ers, kleine zelfstandigen, werkgevers/werknemers in kwetsbare sectoren, zoals de cultuur-, sport- en horecasector	Financiële gevolgen → armoede, toekomstperspectief werkloosheid, positie op de woningmarkt
Kinderen op de basisschool	(Onveilige) thuissituatie en inactiviteit → fysieke en mentale gezondheidseffecten Thuisonderwijs → leer- en ontwikkelingsachterstand
Kinderen op de middelbare school	Beperkingen onderwijs (inclusief stages), minder sociale contacten en minder regie over het leven → leer- en ontwikkelingsachterstanden (Onveilige) thuissituatie en inactiviteit → fysieke en mentale gezondheidseffecten
Leerkrachten	Hoge werkdruk en minder werkvreugde → versombering, angst/zorg, frustratie
Mantelzorgers (zie ook vrijwilligers)	Wegvallen van de reguliere zorg en dagbesteding → angst om dierbaren, hoge werkdruk, relatie met zorgvrager onder druk door toegenomen afhankelijkheid, minder zingeving/voldoening
Mensen met een fysieke en/of mentale (chronische) aandoening	Verschillende COVID-maatregelen → verschillende effecten op de fysieke en mentale gezondheid, het dagelijks leven, de bewegingsvrijheid, sociale contacten, het steunnetwerk, participatie en de toegang tot zorg
Mensen met een fysieke, zintuigelijke, verstandelijke en/ of meervoudige beperking	Verandering in (mantel)zorg, steunstructuren en dagbesteding, afhankelijk van woonomstandigheid → angst, onzekerheid, versombering, vereenzaming, minder participatie in en toegang tot de zorg
Naasten van overledenen	Beperkte aanwezigheid bij afscheid en sterven → verstoorde rouwverwerking/traumatisch verlies
Naasten van bewoners van instellingen voor langdurige zorg	Bezoekbeperking → zorgen en angst vanwege de fysieke en mentale gezondheid van de bewoner
Ouderen	Minder sociaal contact en inactiviteit → versombering, vereenzaming, fysieke en mentale achteruitgang, minder participatie in en toegang tot de zorg
Ouders met jonge (schoolgaande) kinderen	Combinatie van thuiswerken en thuisonderwijs (en soms toegenomen mantelzorg) → druk op thuissituatie, hoge werk- en zorgbelasting
Studenten	Minder sociale contacten en minder regie over het leven → vereenzaming, versombering Minder onderwijs, stagemogelijkheden en (bij)banen → studievertraging, beperkte ontplooiingsmogelijkheden; financiële gevolgen en minder toekomstperspectief
Vrijwilligers	Minder activiteiten en minder dagbesteding → minder voldoening/zingeving, versombering en vereenzaming
Zorgverleners	Hoge werkdruk en andere (benarde) omstandigheden → overbelasting, versombering en *moral injury*

### Lichamelijke gezondheid

COVID-maatregelen beperken enerzijds ziekte en sterfte door SARS-CoV-2-infecties. Anderzijds leiden de maatregelen ook tot ziekte en sterfte. De gehandicaptenzorg, jeugdzorg, thuiszorg, wijkverpleging, geestelijke gezondheidszorg, huisartsenzorg, paramedische zorg en curatieve zorg zijn deels uitgesteld en deels vervangen door zorg op afstand. De omvang van de effecten op zorgbehoeftigen – die vrijwel per definitie verhoogd kwetsbaar zijn – wordt gaandeweg duidelijk aan de hand van evaluaties [2, 4, 5, 7, 9].

Er is sprake van langdurige stress, met mogelijk lichamelijke klachten, een toename van huiselijk geweld en extra middelengebruik [2, 5, 11]. Extra stress ontstaat zowel in de werksfeer door toegenomen werkdruk of juist verlies van werk en inkomen, als in de woonsituatie en recreatieve sfeer vanwege meer verblijf thuis (verveling, isolement), minder sociaal en lichamelijk contact (knuffelen) en minder sport- en recreatiemogelijkheden (ontspanning en zingeving). Sommige groepen zijn extra kwetsbaar, omdat ze al overbelast waren, zoals zorgverleners [2], dan wel omdat ze minder goed kunnen omgaan met een teveel aan stress, zoals mensen met een verstandelijke beperking [7].

### Mentale gezondheid

In de rapporten is vooral aandacht voor de persoonlijke ontwikkeling van jongeren en studenten, en het ‘versomberen’ van de samenleving. Sociaaleconomische gezondheidsverschillen en de kansenongelijkheid in de samenleving worden hierdoor versterkt.

#### Ontwikkeling

Het basisonderwijs is geruime tijd op afstand gegeven en kwam merendeels aan op ouders, met wisselende pedagogische vaardigheden en begeleidingstijd. Het opschorten van het (praktijk)onderwijs voor leerlingen in het voorgezet onderwijs, middelbaar beroepsonderwijs en hoger onderwijs heeft geleid tot studievertraging, verminderde motivatie en extra onzekerheid over werkgelegenheid [2, 6].

Mogelijk nog belangrijker zijn de beperkingen voor de sociaal-emotionele ontwikkeling van jongeren, zowel binnen als buiten school. De effecten laten zich moeilijk voorspellen, maar de relatie van opleidingsniveau, gezondheidstoestand en levensverwachting is welbekend [2, 6]. Kwetsbaar voor leerachterstanden en vervroegde schooluitval zijn vooral gezinnen met een stapeling van risicofactoren, zoals een lage sociaaleconomische status (SES), werkloosheid, beperkte sociale steunstructuren en/of slechte gezondheid [2, 4, 6]. Het achterwege laten van de Cito Eindtoets benadeelde kinderen van lage SES-gezinnen nog eens extra [2].

#### Negatieve emoties

Velen leven in angst en onzekerheid, en experts vrezen een toename van suïcidepogingen [4]. Verschillende groepen zijn om verschillende redenen kwetsbaarder [9]. Zo kan SARS-CoV‑2 extra angst oproepen bij naasten van mensen in zorginstellingen en mensen met bestaande psychische klachten [2, 6, 7]. De onzekerheid speelt ook (niet-gesettelde) jongeren parten [2, 6, 9]. Kwetsbaar zijn vooral groepen waarbij een combinatie van factoren speelt, zoals mensen met een verstandelijke beperking, asielzoekers, en dak- en thuislozen [2, 4, 5, 7].

#### Rouw

Veel COVID-19 patiënten zijn in eenzaamheid gestorven en in de beleving van familie en vrienden ook zonder waardigheid. De beperkingen van het afscheid nemen en deelnemen aan een uitvaart kunnen het rouwproces negatief beïnvloeden [5, 12].

### Dagelijks leven

COVID-maatregelen als *social distancing* en thuiswerken beperken onze bewegingsvrijheid. Het dagelijks leven van wonen, werken, sport, cultuur en recreatie is aan banden gelegd. De openbare ruimte is een ‘schaars goed’ geworden. Sommigen hebben hier meer last van dan anderen. Denk aan stressoren als het begeleiden van schoolgaande kinderen, en het wegvallen van mantelzorgers en formele zorg [2, 3, 7]. Dit kan extra zwaar zijn voor eenoudergezinnen en voor gezinnen met kinderen met gedragsproblemen [2, 9].

Dagbestedingsprogramma’s voor specifieke doelgroepen zijn merendeels weggevallen. Dit raakt zowel cliënten als hun begeleiders, voor een belangrijk deel vrijwilligers. Kwetsbare groepen zijn vooral kinderen/volwassenen met (al dan niet meervoudige) handicaps en/of psychische klachten, en (al dan niet dementerende) ouderen [2, 6, 8, 9].

Sportactiviteiten, feestjes en bezoek zijn al geruime tijd niet meer vanzelfsprekend. Deze activiteiten bieden structuur en sociale contacten, dienen als uitlaatklep en kunnen bijdragen aan de zingeving van het leven [2]. Er is nog weinig zicht op kwetsbare groepen.

### Sociaal-maatschappelijke omgeving

Fysieke en sociale contacten zijn sterk ingeperkt. De financiële situatie, de bestaande (starters- en onderwater)problematiek op de woningmarkt en participatie in de samenleving zijn voor velen verslechterd [5]. Er wordt voor een recessie gevreesd, de steunpakketten en noodregelingen ten spijt. De schuldhulpverlening verloopt moeilijker en er zijn nieuwe groepen met schulden te verwachten, vooral onder starters en mensen op grotere afstand van de arbeidsmarkt [9]. Langdurige werkloosheid en afhankelijkheid van bijstand dreigen, vooral voor kwetsbare sectoren (zoals de cultuur-, transport- en horecasector) en kwetsbare groepen, zoals laagopgeleiden, mensen met een migratieachtergrond, zzp’ers, flexwerkers en mensen met een arbeidsbeperking [9].

De ongelijkheden in de samenleving worden groter. Zo loopt de betaalde arbeidsbijdrage van vrouwen terug ten opzichte van mannen en neemt het aantal werkloosheidsuitkeringen onder jongeren snel toe [2, 6, 9].

De COVID-maatregelen beperken ook de mogelijkheden tot participatie in de samenleving. Asielzoekers en statushouders hebben nog meer moeite hun weg te vinden en zich een plek te verwerven in de Nederlandse samenleving [2]. Dit geldt mogelijk ook voor gedetineerden die na vrijlating terugkeren in een andere maatschappij dan ze kennen en een groter risico lopen op terugval in oude gewoonten [4]. Veel mensen met een verstandelijke beperking verliezen hun dagbesteding of werk (zie hierboven), terwijl dat juist houvast gaf en hielp hun leven te structureren [7]. Ook staat de ondersteuning voor dak- en thuislozen onder druk [9].

## Beschouwing

‘Kwetsbare groepen’ is een containerbegrip. Mensen zijn om verschillende redenen extra kwetsbaar voor COVID-maatregelen. Dit heeft consequenties voor de behoeften aan en mogelijkheden voor zorg en ondersteuning. Specificaties zijn nodig om het zorg- en ondersteuningsaanbod vorm te geven en te optimaliseren, waarbij er expliciete aandacht moet zijn voor risicocumulatie en toename van weerbarstige sociaaleconomische gezondheidsverschillen en kansenongelijkheid in bepaalde groepen [17].

Elf adviesrapporten zijn nageplozen om zicht te krijgen op extra kwetsbare groepen voor COVID-maatregelen. Sommige kwetsbare groepen zijn breed omschreven, zoals ouderen, en omvatten meerdere subgroepen en neveneffecten, bijvoorbeeld verminderd zelfredzame ouderen die beperkingen ondervinden in het dagelijks leven en ouderen met een beperkt sociaal netwerk die lijden onder de inperking van sociale activiteiten. Andere groepen zijn juist smal omschreven en gerelateerd aan één neveneffect, zoals rouwverwerking door naasten van overledenen of participatie in de samenleving door ex-gedetineerden. Bij sommige groepen is al een stapeling van neveneffecten zichtbaar (zie tab. 1), bijvoorbeeld middelbare scholieren en studenten voor wie ontwikkelingsmogelijkheden, inkomen (veelal flexwerkers) en het sociale leven deels wegvallen. Of mensen met een verstandelijke beperking die meer gevolgen ondervinden van het wegvallen van werk of dagbesteding en de inperking van sociale contacten. Bij andere groepen zijn stapelingseffecten minder zichtbaar, maar evengoed te verwachten, zoals asielzoekers, ouderen, dak- en thuislozen of jonge vrouwen.

De rapporten bieden een eerste uitsnede van de neveneffecten van en kwetsbare groepen voor COVID-maatregelen. Dit maakt een professionele discussie mogelijk over de concretisering van de zorg en ondersteuning aan kwetsbare groepen. Verdieping en verrijking is natuurlijk nodig. We hebben alleen Nederlandse adviesrapporten beoordeeld, die een nog geringe ervaringsbasis hebben en op veel aannamen zijn gebaseerd. Zo moet nog blijken in hoeverre jongeren een leer- en ontwikkelingsachterstand oplopen en in welke mate steunmaatregelen economische onzekerheden compenseren. De adviesrapporten vormen een momentopname en zullen snel gedateerd raken. Ook zullen nieuwe maatregelen gepaard gaan met nieuwe kwetsbare groepen. Zo dreef de anderhalvemetersamenleving blinden en slechtzienden in het nauw en versterkt de mondkapjescultuur inmiddels de isolatie van doven en slechthorenden. Sommige thema’s in het maatschappelijke debat blijven onderbelicht in de adviesrapporten, zoals de coronakilo’s (door het sluiten van de sportscholen), spirituele aspecten (beperkingen voor religieuze bijeenkomsten en hoogtijdagen, zoals geboorte en huwelijk) en milieueffecten. Ook ontbreekt aandacht voor bepaalde kwetsbare groepen, zoals gevangenen (isolatie, het wegvallen van de dagbesteding), illegalen en slachtoffers van mensenhandel (zorgdrempels), die al wel door Douglas et al. waren benoemd [16].

De positieve neveneffecten van de COVID-maatregelen zijn in deze analyse buiten beschouwing gelaten. Zo hebben we geen aandacht besteed aan het feit dat driekwart van de mensen met psychoses juist minder problemen hebben ervaren [8], aan de toegenomen aandacht voor gezonde voeding en levensstijl, of aan de stimulans om nieuwe werkvormen en banen te creëren. Om afwegingen tussen risico’s en risicogroepen te kunnen maken zijn aanvullende methoden nodig, zoals multicriteria-analyses, maatschappelijke kosten-batenanalyses of welvaartsanalyses.

Een checklist met effecten op het ‘(samen)leven’ is een goede eerste stap om de neveneffecten van COVID-maatregelen te categoriseren. De WHOQOL helpt om ‘in de buurt te komen’ van de doelstelling: snel zicht krijgen op extra kwetsbare groepen om tijdens en na een (infectieziekte)crisis de professionele discussie over zorg- en ondersteuning te voeden. De WHOQOL kent echter ook enkele beperkingen. Zo overlappen en beïnvloeden de domeinen elkaar. ‘Werk’ valt bijvoorbeeld onder ‘zelfstandigheid’, maar ‘financiën’ onder ‘omgeving’. Verder beïnvloeden de lichamelijke, mentale en sociale gezondheidsaspecten elkaar onderling. Deze interacties worden niet direct inzichtelijk door screening van documenten via de WHOQOL. Het vraagt ook verdieping om zicht te krijgen op individuele variaties in kwetsbaarheden, coping-vaardigheden en veerkracht binnen groepen, wat essentieel is voor het organiseren van passende zorg en ondersteuning [18].

## Conclusie en aanbevelingen

De WHOQOL lijkt, ondanks de hier beschreven beperkingen, bruikbaar om een eerste systematisch overzicht te krijgen van de neveneffecten van de COVID-maatregelen. Analyse van elf rapporten maakt diverse bekende en nieuwe kwetsbare groepen zichtbaar. Patiëntverenigingen, belangenbehartigers en brancheverenigingen hebben verschillende signalementen en handreikingen uitgebracht waarmee de lijst kan worden verrijkt (groeimodel). Een overzicht van kwetsbare groepen biedt een basis om de monitoringsactiviteiten en informatievoorziening aan te scherpen, en waar nodig de contacten tussen (verondersteld) kwetsbare mensen, zorgverleners, GGD’en, sociale wijkteams en overheden te versterken. Naarmate de groepen en hun kwetsbaarheden duidelijker in beeld komen, kan beter maatwerk worden geleverd. Denk aan het aanbieden van passende informatie over lichamelijke gezondheid en mentaal welbevinden, het organiseren van praktische hulp en het leveren van emotionele of financiële ondersteuning.

De WHOQOL-vragenlijst kan, eventueel in aangepaste vorm, bijdragen aan verdiepend onderzoek binnen en tussen groepen die verhoogd kwetsbaar lijken voor de negatieve neveneffecten van COVID-maatregelen, en om de (langetermijn)effecten van COVID-19 te categoriseren. Wanneer we beter zicht hebben op kwetsbare groepen en specifieke kwetsbaarheden kunnen we in cocreatie inschatten welke mensen daadwerkelijk in de problemen komen en op welke manier zij het beste met passende ondersteuning kunnen worden bereikt. Dat vergt een netwerkinspanning van veel partijen: kwetsbare mensen zelf, zorgverleners en leerkrachten, andere professionals en overheden. Kwetsbare groepen dragen vrijwel per definitie de zwaarste lasten van rampen en crises. Door de handen ineen te slaan kunnen we de onevenredig zware last van COVID-maatregelen voor kwetsbare groepen enigszins met en voor hen beperken. Net zoals we dit proberen te doen voor groepen die verhoogd kwetsbaar zijn voor COVID-19.
